# The Interplay between ESCRT and Viral Factors in the Enveloped Virus Life Cycle

**DOI:** 10.3390/v13020324

**Published:** 2021-02-20

**Authors:** Bo Meng, Andrew M. L. Lever

**Affiliations:** Department of Medicine, Biomedical Campus, University of Cambridge, Cambridge CB2 0AW, UK

**Keywords:** ESCRT, budding, enveloped virus, late domain, NEDD4, retrovirus

## Abstract

Viruses are obligate parasites that rely on host cellular factors to replicate and spread. The endosomal sorting complexes required for transport (ESCRT) system, which is classically associated with sorting and downgrading surface proteins, is one of the host machineries hijacked by viruses across diverse families. Knowledge gained from research into ESCRT and viruses has, in turn, greatly advanced our understanding of many other cellular functions in which the ESCRT pathway is involved, e.g., cytokinesis. This review highlights the interplay between the ESCRT pathway and the viral factors of enveloped viruses with a special emphasis on retroviruses.

## 1. Introduction

**Figure 1:**
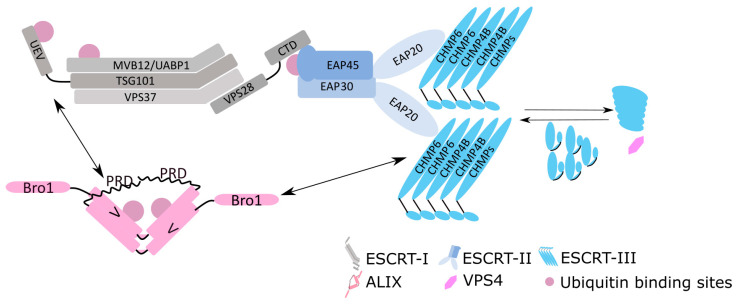
Schematic diagrams of the mammalian core ESCRT protein complexes. Direct contacts are made to show the interaction among the ESCRT components e.g., VPS28 and EAP45, and EAP20 and CHMP6. Double-headed arrows denote the intermolecular interactions between ALIX and TSG101 and ALIX and CHMP4B.

In the 1990s, a series of published studies on the major retroviral structural protein Gag identified regions whose disruption completely arrested virus release from infected cells; the so called ‘late’ stage of their life cycles [1,2,3,4]. The viral protein motifs involved acting at this period of assembly and budding were termed “late domains” (L domains). Identification of these critical regions spurred investigation into finding their cellular binding partners. Two independent yeast two-hybrid studies using HIV-1 (human immunodeficiency virus type 1) Gag-p6 as bait identified TSG101 as the host interacting factor [5,6], which was later also shown to bind HIV-2 Gag [7]. Vps23, the yeast orthologue of mammalian TSG101, had been previously implicated in degrading surface proteins in yeast and in mammalian cells [8,9]. Dominant negative versions of VPS4, an AAA-ATPase required for formation of multivesicular bodies (MVB) [10], were also shown to block virus budding [5]. Together, these studies revolutionised our understanding of how HIV hijacks the host endosomal trafficking pathway to bud away from infected cells. Subsequent systematic screening of human homologues of class E genes in yeast confirmed similar intermolecular and intramolecular interactions [11,12] (Figure 1), corresponding to the then newly identified ESCRT-I (endosomal sorting complexes required for transport) complex in yeast [13] and revealed a remarkable parallel in how pathogens utilise these host factors mechanistically to pinch off from their budding sites. Other viruses were later shown to use the same pathway [5,14,15]. Some non-enveloped viruses also utilise the ESCRT system to form their replication complexes (e.g., Carnation Italian ringspot virus (CIRV [16]), tomato bushy stunt virus (TBSV [17]), and brome mosaic virus (BMV [18])) and for virus release (e.g., Hepatitis A and Chikungunya viruses [19,20]). Here, however, we focus on how enveloped viruses, with a particular emphasis on retroviruses, hijack the ESCRT pathway for the purposes of viral export. The broader cellular functions of the ESCRT pathway are extensively reviewed elsewhere [21,22,23].

## 2. Overview of the ESCRT Pathway

The ESCRT system, originally discovered in yeast [24,25], where its disruption caused a severe defect in vacuolar sorting, is conserved from the archaea throughout eukaryotes [26]. The core ESCRT system is composed of ESCRT-I (TSG101 (Vps23), VPS28, VPS37, and MVB12/UBAP1). Nomenclature of ESCRT components in metazoans is in upper case letters and, for yeast, capitalized lowercase letters (examples in brackets), thus for ESCRT-II: EAP20 (Vps25), EAP30 (Vps22), and EAP45 (Vps36) and ESCRT-III: IST1 and CHMP1–7 (charged multivesicular protein; Figure 1). ESCRT-I structurally forms an elongated hetero-tetramer [27,28] and links to HRS (hepatocyte growth factor–regulated tyrosine kinase substrate), a component of the ESCRT-0 HRS/STAM complex, through TSG101 binding to a PSAP (Pro-Ser-Ala-Pro) domain [29,30]. ESCRT-II is also a hetero-tetrameric complex composed of one copy of EAP45 and EAP30 and two copies of EAP20 forming a ‘Y’ shaped structure [31,32]. The linkage between ESCRT-I and ESCRT-II is through the C terminus of VPS28 and the N terminus of EAP45 [28,33,34]. Engagement of ESCRT-III to ESCRT-II is through CHMP6 and EAP20. There are 12 mammalian ESCRT-III like proteins (CHMP1A/B, 2A/B, 3, 4A/B/C, 5, 6, and 7 and IST1) showing similar coiled-coil structural conformations [35,36]. Most of the ESCRT-III proteins exist in an autoinhibited “closed” formation in the cytoplasm [37,38] and are polymerised into various filamentous structures capable of deforming membranes both in vitro and in vivo [39,40,41,42,43]. It is proposed that the polymerisation and depolymerisation of ESCRT-III provides the driving force for membrane scission [43,44,45]. The ESCRT-associated protein ALIX (Bro1 homologue in yeast) consists of N terminal Bro1 and C terminal proline-rich domains (PRD) flanking a ‘V’-shaped central domain. ALIX recruits CHM4B via its Bro1 domain providing an alternative access route to ESCRT-III [46,47,48]. Final membrane scission is triggered by the hexametric ring of AAA-ATPase VPS4 [49], which arrives after ESCRT-III recruitment. VPS4 hydrolyses ATP providing the energy for thinning the budding neck as well as depolymerising and recycling the ESCRT-III polymer through its central hole (for reviews on membrane scission, refer to References [36,50]). It is not yet fully understood whether VPS4 also plays a role in membrane remodelling as well as scission. In addition to its involvement in sorting ubiquitinated cargos into MVB where the name ESCRT was first coined, ESCRT is involved in many other cellular functions where scission of a negative membrane curvature “bud” occurs. Important examples include cytokinesis [51,52], nuclear envelope sealing [53,54], endo-lysosomal membrane repair [55], plasma membrane repair [56], and autophagy [57]. Membrane scission-independent ESCRT functions have also been documented to involve transcriptional control [58,59,60,61] and RNA regulation and trafficking [62,63,64].

## 3. Viral Factors Involved in Entry to the ESCRT Pathway

### 3.1. Late Domains

The tetrapeptide motif PT/SAP (Pro-Thr/Ser-Ala-Pro) was first described in the p6 domain of the HIV-1 Gag protein (a polyprotein consisting of MA, CA, NC, and p6 with two short peptides between CA and NC and between NC and p6) and later identified in other retroviruses including HIV-2 and simian immunodeficiency virus (SIV), and, in other virus families, including arenaviruses, filoviruses, rhabdoviruses, and reoviruses (for a comprehensive list of known late domains, refer to Reference [65]). The PT/SAP motif functions through the binding to the N terminal UEV domain of TSG101 [66]. Mutations perturbing late domain functions are observed in subtype C HIV-1 infected, treatment-naive patients in which a duplication of the PTAP domain occurs, resulting in a higher affinity for TSG101 [67]. In contrast to subtype C, subtype B viruses bearing a similar PTAP duplication together with mutations found in the viral protease increase their replication fitness through effects on Gag proteolytic cleavage rather than increasing the efficiency of viral release [68]. The PPXY sequence (Pro-Pro-X-Tyr; where X refers to any amino acid) was first identified as important in Rous sarcoma virus (RSV) [4] and, subsequently, shown in other retroviruses including murine leukemia virus (MLV), Mason-Pfizer monkey virus (M-PMV), and human T-cell leukemia/lymphoma virus type 1 (HTLV-1) as well in the filoviruses, arenaviruses, rhabdoviruses, and hepadnaviruses. The PPXY motif plays a key role in recruiting host WW domain-containing proteins [4,69], which are implicated in ubiquitinating viral and/or cellular factors for access to the ESCRT pathway (see below). The YPXL (Tyr-Pro-X-Leu; where X refers to any amino acid) domain was first identified in equine infectious anaemia virus (EIAV) [70]. Subsequent research into the budding of SIV and HIV showed ALIX is packaged into the virion via a YPXL domain, which is also located in the p6 domain of Gag [12]. Many virus families encode YPXL domains including arenaviruses and flaviviruses. The YPXL motif functions through binding to the V domain of ALIX [46]. HIV-1 subtype C virus naturally lacks a YPXL domain [71], but a PYXE insertion has evolved in treatment-experienced subtype C infected patients [72]. The effect of these mutations is to enhance the virus replication in the presence of antiretroviral therapy (ART) by allowing engagement with ALIX, which, otherwise, does not occur [72,73,74]. Crystallographic studies show both PYXE and YPXL motifs share a similar mode of binding to ALIX [75]. Very recently, a PLPPV (Pro-Leu-Pro-Pro-Val) sequence motif located in the p8 region of mouse mammary tumour virus (MMTV) Gag has been described to provide late domain activity [76]. Introduction of this motif also rescues the budding defect of EIAV lacking an authentic YPXL domain. The PLPPV domain thus potentially represents a new class of late domain in the retrovirus family, even though its cognate cellular binding partner remains unknown.

In addition to the late domains identified within the retrovirus family, an FPIV (Phe-Pro-Ile-Val) sequence motif located in the M proteins of paramyxoviruses has been shown to be required for viral budding [77,78]. The FPIV motif can functionally rescue an HIV-1 budding defect caused by the lack of a PTAP domain.

HIV, HTLV, Ebola, LCMV (lymphocytic choriomeningitis virus), and MLV encode multiple L domains while EIAV only has one. The interchangeability of different classes of late domains demonstrated their main roles are to serve as docking sites for host factor recruitment [4,70,79]. In viruses with more than one type of late domain, there appears to be a hierarchy of importance. For example, ALIX recruitment via a YPXL domain is not essential in HIV-1 budding if a PTAP-TSG101 interaction is present. However, a recent study showed that ALIX is required for the efficient engagement of CHMP4B and VPS4 [80]. Multiple late domains may also play distinct roles at different stages of the assembly of the same virus. For example, the PPPY and PSAP dual bearing Gag in M-PMV [81] and HTLV-1 [82,83,84] play roles at early envelopment and membrane scission, respectively. It is thought the presence of multiple late domains and their differential reliance in various cell types may provide an evolutionary advantage to facilitate virus spreading.

### 3.2. Ubiquitination

In addition to the late domain acting as a critical entry point for the ESCRT system, ubiquitination might provide an alternative route to this, analogous to sorting of ubiquitinated cargos into MVB. TSG101, UBAP1, EAP45, and ALIX all contain ubiquitin binding sites (Figure 1). The observation that ubiquitin is packaged into virions and that Gag is ubiquitinated in virions suggested ubiquitin plays a role in retroviral budding [85]. Depletion of cellular ubiquitin by adding proteasomal inhibitors decreased budding efficiency, implying that the abundance of cellular ubiquitin pool positively impacts virus budding [85,86,87]. Consistent with these observations, direct ubiquitination of a late domain defective EIAV rescued virus release [88] and a deubiquitinase conjugated Gag in HIV-1 failed to bud [89]. In contrast, ubiquitination in Gag is not required in PPXY mediated prototypic foamy virus (PFV) budding, where ubiquitin acceptors in Gag are largely absent [90,91].

RSV was the first virus to be shown to have a PPXY motif that directly recruits members of the NEDD4 ubiquitin E3 ligase family whose catalytic activity is required for efficient RSV budding [92,93]. NEDD4 and, in particular, its WW and HECT domains are required to facilitate Gag assembly of HTLV-1 [83,94,95]. Similar findings are also observed in MLV whereby the HECT ubiquitin E3 ligase is required for promoting the budding of MLV and is reliant on the PPXY motif and the catalytic domain of the HECT ligase [96]. Later, it was shown that arrestin-related traffic proteins (ART) are recruited to the budding site and, potentially, serve as adaptors to recruit both HECT ligases and ESCRT components [97]. Filoviruses (e.g., Ebola and Marburg [98]) and rhabdoviruses (e.g., vesicular stomatitis virus (VSV [99])) also encode a PPXY motif in their VP40 and M proteins, respectively, that specifically interacts with WW domain containing proteins to facilitate viral release. Arenaviruses also encode a PPXY domain in their matrix Z protein [100], through which the NEDD4 ligase is recruited [101,102]. However, ubiquitination of the PPXY-containing Z protein of LCMV itself is insufficient for virus budding, suggesting factors other than Z is involved [101].

HIV does not encode a PPXY motif and yet overexpression of both NEDD4-1 and NEDD4-2 (also known as NEDD4L) ubiquitin E3 ligase rescues a PTAP budding defect in HIV-1 [103,104,105]. NEDD4-2 could also rescue a PTAP and YPXL double mutant whereas NEDD4-1 could not. It was later shown that an adaptor protein AMOT was involved in bridging Gag and NEDD4-2 [106] to ubiquitinate viral or cellular factors in the proximity of the assembling Gag, facilitating its access to the ESCRT pathway via its recognition of ubiquitin [106,107]. This neatly explains why NEDD4-2, which binds to PPXY that HIV lacks, rescues the late domain mutant lacking both PTAP and YPXL domains [103]. AMOT-1 was also shown to facilitate paramyxovirus budding in a similar manner as it does in HIV [108,109]. However, AMOT-1 (but not the other members of the agiomotin family) is specifically required for bridging the M protein and E3 ubiquitin ligase [109]. In contrast to NEDD4-2 mediated rescue of a crippled PTAP virus through its binding to AMOT, NEDD4-1 coimmunoprecipitates with and ubiquitinates ALIX [105]. Endogenous NEDD4-1 is required for ALIX-mediated rescue of a PTAP mutant virus, suggesting NEDD4-1 is recruited to the budding site. Additionally, this functional rescue requires ALIX, YPXL, and NC interactions.

Despite no identified late domains in HCV as described above, its NS2 is ubiquitinated and interacts with HRS via its ubiquitination interacting motif (UIM) and, in doing so, gains access to the ESCRT pathway [110].

## 4. Early Acting Components

### 4.1. ESCRT-I

**Figure 2:**
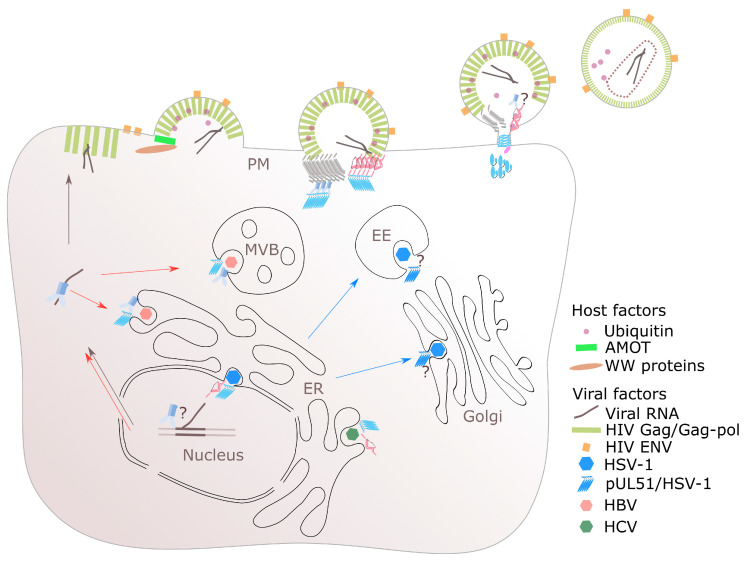
Multifaceted roles of ESCRT in the enveloped virus life cycles. The extracellular virus budding at the plasma membrane (PM) is exemplified with a late assembly and budding event of HIV. HIV, and other enveloped viruses, e.g., filovirus, rhabdovirus, and arenavirus (not shown), also utilises ubiquitination in gaining access to the ESCRT system for virus export. HSV-1 is unique as two steps of envelopments occur with the requirement of ALIX (ALG2-interacting protein X) and ESCRT-III for the nuclear or primary envelopment while a unique viral encoded ESCRT-III-like protein pUL51 is thought to be involved in membrane constriction for the secondary envelopment. ESCRT-II is involved in ribonucleoprotein trafficking for both HIV and HBV. The color scheme is the same as those in Figure 1 unless stated otherwise. Question marks denote the possibility of the involvement of ESCRT or other factors at various stages of virus life cycles. AMOT: angiomotin. ER: endoplasmic reticulum. EE: early endosome. MVB: multivesicular body.

Interactions between the components of ESCRT-I and the late domain-containing viral factors are the main entry point to the ESCRT pathway (Figure 2). Functional disruption of a PTAP and TSG101 interaction by either siRNA knockdown or mutagenesis or expression of a truncated form of TSG101 recapitulates a defective HIV budding phenotype [5,111,112]. Depletion of UBAP1 or MVB12, however, did not affect HIV release [113,114], but infectivity in the latter was reduced due to the malformation of immature virus particles [114]. VPS37 fusion Gag did rescue a crippled PTAP budding, indicating it functions in HIV budding [115,116]. VPS28 binding to TSG101 is known to be required for HIV budding [116,117]. Recently, the crystal structure of the core of human ESCRT-I was resolved, showing a helical filamentous structure mediated by VPS28 [118]. Ablating the residues in VPS28 that are involved in this interaction completely abolishes this helix formation in vitro and results in failure of autophagophore closure and HIV release. This has opened up the exciting possibility that ESCRT-I is not merely required for cargo sorting and linking to downstream of ESCRT components as previously thought, but may serve as a nucleation platform where membrane remodeling and fission complexes are recruited.

### 4.2. ESCRT-II

Canonical linkage of ESCRT-I to III occurs via the intermediate ESCRT-II. ESCRT-II was identified through mutations in a subset of genes that cause class E defects in yeast whose gene products were found to form heterotetrameric complexes that co-migrate as a complex. This complex is comprised of Vps36, Vps25, and Vps22, which was later classified as the ESCRT-II complex [119]. Remarkably, an independent line of research on mammalian transcription factors isolated from rat liver extract found a protein complex associated with ELL (Eleven-Nineteen Lysine-Rich Leukemia gene, an elongation factor associated with RNA polymerase II) that also co-migrates and is required for de-repression of transcriptional initiation [58,59]. This ELL associate protein (EAP) complex is comprised of three proteins of molecular weights of 20, 30, and 45 kDa that turned out to be orthologues of the previously mentioned ESCRT components in vacuolar sorting in yeast. The ESCRT-II complex has interactive domains for ubiquitin [120], ESCRT-I [33], and specific membrane lipid components [33,120].

It was proposed that ESCRT-II is important in linking ESCRT-I and ESCRT-III for endosomal cargo sorting [43,119], but the engagement of ESCRT-II in virus release has been controversial, initially being regarded as dispensable in HIV-1 [121,122]. The lack of a virus budding defect in early siRNA knockdown studies of ESCRT-II was explicable by an alternative link between VPS28 and CHMP6 for access to ESCRT-III [123]. However, in contrast, in vitro reconstitution experiments using giant unilamellar vesicles demonstrated that ESCRT-II was involved in linking to ESCRT-I at HIV Gag forming clusters and was required for the recruitment of ESCRT-III [124]. Thus, the early siRNA knockdown studies might have been confounded by residual amounts of protein that survived the treatment but was still present in adequate amounts to facilitate budding. To reconcile these discrepancies, the role of ESCRT-II was revisited in a complete CRISPR/Cas9 EAP45 knockout (KO) model cell line (HAP1) and in HIV permissible T cells [61,125]. Here, it was demonstrated that, upon complete removal of EAP45, there was a profound decrease in virus release and of virus spread in T cells. The defect was specific to EAP45 since this phenotype was rescued in KO cells by expressing *in trans* the constructs containing the N terminal region of EAP45, supporting that the linkage to ESCRT-I is required for HIV release in this system [125]. EAP45 has also been visualised in proximity to Gag clusters and the co-localisation was also dependent on its N terminus (Meng et al. submitted), in accordance with the biochemical evidence. Coincidentally, this reevaluation joins emerging evidence on cytokinesis in which involvement of ESCRT-II was also previously considered unnecessary [51,52], but in which it has since been confirmed to play an integral role [126,127].

In contrast to studies on virus budding, ESCRT-II’s role in transcription has been largely unexplored. From the studies on EAP45 KO cells, it was clear that, in addition to the impaired budding, the abundance of HIV transcripts was drastically reduced [61]. This decrease at the RNA level was not due to impaired proviral integration [125]. EAP30 (but not EAP20 or EAP45) forms a complex with IRF3 and its transcriptional coactivator CBP (CREB binding protein) in a virus-induced manner, which, in turn, drives antiviral gene expression [60]. Despite the scarcity of research in this area, it is tempting to speculate that, given the roles of ESCRT-II in transcriptional regulation of cellular genes, it would not be surprising if ESCRT-II as a whole or its constitutive individual components were also involved in viral gene expression. However, this awaits further investigation.

The versatility of the ESCRT-II complex also extends to ribonucleoprotein (RNP) trafficking, independent of its functions in endosomal sorting and transcriptional regulations. This was first shown in the Drosophila oocyte where VPS36 binds *bicoid* RNA in a sequence-specific manner and this binding is required for the correct localisation of RNA at the anterior pole of the oocyte [63]. Intriguingly, only ESCRT-II within the ESCRT pathway is required for correct targeting of *bicoid* RNA, suggesting there may be a unique role for ESCRT-II in RNA regulation and trafficking. ESCRT-II was later reported to bind various mRNAs in *Xenopus laevis* eggs, highlighting that this activity is conserved across different species [64]. Viruses also usurp ESCRT-II for trafficking of viral RNA. EAP30 is involved in viral RNA trafficking in HIV-1 [62]. Depletion of ESCRT-II reduced the level of migration of gRNA from the nucleus to the cytoplasm. In HBV, functional ablation of EAP30 and EAP45 did not affect the total level of HBV-specific mRNAs but did reduce the level of packaged pregenomic RNA (pgRNA), suggesting the trafficking of pgRNA is affected similarly to what is reported in HIV-1 [128].

### 4.3. ALIX

ALIX is a versatile cellular protein involved in many cellular functions through its binding to different effector proteins such as TSG101 [11,12], CEP55 [51,129], tyrosine kinase [130], endophilin [131], and syntenin [132,133]. The involvement of ALIX in the dual PTAP-YPXL domain bearing retroviruses is largely considered secondary or redundant. However, overexpression of ALIX rescues the budding defect of a PTAP mutant and this rescue effect is independent of its binding to TSG101 but requires access to YPXL and CHMP4B [46,47] and intact ubiquitin binding sites in the ALIX V domain [134]. In addition to a YPXL-ALIX interaction, the NC component of Gag also binds ALIX through its Bro1 domain [135,136] with RNA possibly providing a bridging role [137]. It was shown by Bouamr and colleagues that NC mimics the PDZ domains of syntenin, which is a host cellular protein that functions in membrane dynamics and cell signalling, in targeting Gag to the plasma membrane [133]. A functional exchange of NC with PDZ rescues a PTAP budding defect upon overexpressing ALIX. Bouamr et al. proposed that a subset of the Gag polyprotein NC domains at the budding neck trades off RNA for membrane-binding in a similar manner as MA targeting Gag to the lipid raft at the viral assembly site [138], which could physically unleash YPXL in recruiting ALIX. This attractive model potentially explains why both NC and YPXL, which are physically located adjacent to each other in Gag, play an interdependent role in virus release [135,136]. In vitro studies seem to favour this hypothesis in that structurally p6 has been shown to fold back against NC [139] and p6 bearing Gag appears to have a selective advantage in packaging un-spliced (genomic) viral RNA over cellular or spliced viral RNA [140]. Although the nature of such an RNA ‘trade’ is unknown, it is conceivable that this model also provides a possible mechanism for the quality control of the packaged specific viral RNA as the spatiotemporal disruption of the budding process either through NC [135,136,141,142], p6 [1,2,143], or viral RNA [144] mutation leads to production of the non-infectious immature viruses, most likely through the mistiming of the viral protease activation within the bud [145].

Apart from HIV budding at the plasma membrane, herpesviruses undergo two steps of envelopment: primary envelopment and secondary envelopment. Primary envelopment occurs in the nucleus where the nucleocapsid is engulfed by the inner nuclear membrane (INM) and protrudes into the perinuclear space before de-envelopment and release to the cytosol. The NEC (nuclear egress complex), itself capable of deforming the membrane [146,147,148], is comprised of conserved herpes viral homologues to pUL31 and pUL34 of herpes simplex virus 1 (HSV-1), forming a heterodimer anchored to the INM by pUL34 [149]. This, coupled with the observations that the number of particles in the nucleus is indistinguishable in the presence of dominant negative VPS4 [150,151], suggested ESCRTs are not involved in nuclear envelopment. However, recent evidence shows ALIX and CHMP4B bind to NEC and this binding is necessary and sufficient for nucleocytoplasmic export [152]. This is not unprecedented as BRF1 of EBV, which is the homologue to pUL34, also co-immunoprecipitates with ALIX [153,154]. Despite the discrepancy on the primary envelopment, neither ALIX nor TSG101 is required for the secondary envelopment [155,156,157,158].

### 4.4. Formation of Early ESCRT Assemblies

Recent structural studies have revealed the core of ESCRT-I and the C terminus of ALIX form filaments in vitro [118,159]. Strikingly, what appears to be the same filaments are seen in the form of “spoke-like projections” decorating the inside of the budding neck [160]. Live-cell imaging studies show that TSG101 is recruited as Gag multimerises while a flux of ESCRT-III and VPS4 are recruited predominantly at a later time point after the Gag signal reaches a plateau [161,162,163]. These observations support a notion that the early ESCRT components may also assemble as a modular platform for the recruitment of the downstream ESCRT factors, in contrast with the conventional viewpoint of them being a mere target entrance point. ESCRT-II forms a part of the membrane curvature sensing complex [164,165], whose interaction with ESCRT-I is required in cytokinesis [126] and in HIV budding [125]. It is, therefore, conceivable that ESCRT-II may be recruited onto this platform. This recruitment could, however, depend on the spatiotemporal invagination process of the bud as the linkage to ESCRT-I becomes more important with Gag/Pol (a larger polyprotein synthesised due to a frameshift at the 3’ coding region of *gag*) but not Gag only [125]. Hypothetically, ESCRT-II could be recruited either as a preformed multimer with ESCRT-I or after the ESCRT-I platform is formed at the budding neck. Data from the live-cell imaging studies of ESCRT-II in HIV budding show the co-occurrence of Gag and the ESCRT-II at the budding site, but this co-occurrence is highly dynamic, reminiscent of different phenotypes of recruitment of other ESCRT factors that have been observed in the system (Meng et al. submitted). More studies defining the kinetics of ESCRT-II recruitment in the process of Gag multimerisation together with the other early and late ESCRT components would be desirable to address this question. Alternatively, ESCRT-III could directly engage onto this platform via CHMP6 and VPS28 and/or TSG101 and ALIX.

Other docking assemblies may exist [166]. The multifaceted roles of ALIX in interacting with various viral and cellular factors suggest it could be one such [167]. Importantly, the PRD domain of ALIX reversibly forms fibrils in vitro and the disassembly of this polymer may be regulated by phosphorylation [159]. Based on this observation, it is possible that the polymerisation of ALIX triggered by its PRD domain may concentrate Bro1 and CHMP4 interactions above the threshold that is required for membrane scission. The recruitment of ALIX during HIV-1 budding shows a dynamic burst once Gag multimerisation is completed [168], analogous to the late ESCRT acting components and unlike the progressive recruitment shown by TSG101 (3–5 min). This observation seems to argue against the formation of ALIX assemblies at the early stage of virus assembly. However, it remains possible that a rapidly progressive recruitment of ALIX and CHMP4B (within 10 s) occurs when the Gag shell is fully completed [169]. In EIAV, an apparently simpler retroviral budding model, a streamlined sequential recruitment of ESCRT components is observed triggered by a sole YPXL and ALIX interaction [170]. In this model, ALIX is recruited in a progressive manner as Gag multimerises (5–20 min) [163]. This also suggests ALIX could act early and form such similar assemblies as observed in the ESCRT-I core.

## 5. Late Acting Components/ESCRT-III and VPS4

Despite different viruses using different subsets of ESCRT complexes to bud from the infected cells, convergence at the recruitment of ESCRT-III for membrane scission is a common signature. Studies on HIV-1, MLV, and EIAV showed that only a subset of ESCRT-III proteins are required, with CHMP2 and CHMP4 being particularly important [170,171,172]. CHMP3 and CHMP6 are colocalised to Gag puncta in a biochemical reconstitution system in HIV-1 [124], even though the siRNA knockdown of these factors only gave a two-fold reduction in virus release at most [41,172]. This highlights that there is redundancy between members of CHMP proteins in ESCRT-mediated virus budding.

The involvement of VPS4 in HSV-1 (a member of alpha-herpesvirus) assembly is clear [150]. However, the requirement for VPS4 in beta-herpesvirus HCMV is controversial with conflicting results on its involvement [155,156]. Recently, using inducible cell lines expressing various ESCRT-III components, Streck et al. [173] demonstrated that neither ESCRT-III nor VPS4 is required for the production of infectious HCMV virions, despite the clear presence of VPS4 at the assembly site [155,156,173]. The formation of a narrow bud neck in HCMV secondary envelopment was observed with a minimal distance of 9.1 nm between membranes [174], and yet no electron-density was observed within this gap in contrast to the budding neck in HIV-1 [160]. pUL71 underlines the neck with a predicted ESCRT-III-like structure [173], but the structural information first came to light on its homologue pUL51 in HSV-1 [175]. pUL51 is adopted as helix-turn-helix conformation and forms filaments *in vitro*, highly resembling the classic ESCRT-III structure [175]. Despite its conservation throughout all herpesviruses and its cellular localisation in the budding viruses, pUL51 is unlikely solely accounts for cytoplasmic envelopment as ablation of this did not completely abolish the viral release [175,176]. Given the ability of membrane deformation [174] and formation of the ESCRT-III-like structure in vitro [175], it is possible that, together with the yet undefined cellular or viral factors, pUL71/pUL51 is likely involved in constricting the budding neck and the subsequent scission event.

Other membranous structures apart from those at the endosome and the plasma membrane, such as the peroxisome and endoplasmic reticulum (ER), are also utilised for the formation of the replication complexes by non-enveloped viruses, such as TBSV and BSV [17,18]. Japanese encephalitis virus (JEV) and dengue (DENV) also form replication complexes on the ER. Depletion of a subset of ESCRT-III did not affect the formation of such complexes, but particle release was decreased [177]. Depletion of VPS4, however, did not affect virus production, unlike what is otherwise, universally observed in ESCRT-dependent viral budding events. This suggests the existence of other unknown factor(s) whose function is similar to VPS4, which is involved in this process. ESCRT is required during the HCV life cycle, but consensus has not yet been reached as to what stage of its life cycle this involves. Some reports using the dominant negative ESCRT components or siRNA knockdown demonstrated the involvement of ESCRT-III and VPS4 in HCV release [178,179]. Others, however, show that the virus assembly is affected [110]. ALIX depletion, however, specifically rescues virus release disproportionally [110], which has also been documented in yellow fever virus [180]. Taken together, studies in flavivirus show the perturbation of ESCRT does not affect the flavivirus replication complex formation and genome replication, but the requirement of ESCRT-III at the late stage of virus assembly/release is conserved.

## 6. Conclusions

Learning how the ESCRT system is hijacked by viruses has greatly advanced our knowledge in understanding its normal cellular functions including cytokinesis and nuclear envelope sealing. Two examples among many that have been revolutionised from the research into this field. The multiple entry sites and the apparent internal redundancy of its components highlight the extreme flexibility of this ancient machinery. Viruses across different families have evolved to target a variety of routes of entry into the system, ultimately resulting in a functional convergence to ESCRT-III. The ESCRT system is positioned to spatiotemporally choreograph the quality control of viral budding and possibly the capture of the genome. Viral RNA must be packaged to produce infectious virus particles, but to what extent ESCRT is involved in this process remains to be firmly established. Despite great advances in our comprehension of the ESCRT-dependent budding process, the functions of ESCRT components in viral replication have yet to be completely defined. The fact that ESCRT demonstrates functional redundancy in its pathways for cellular processes opens up the possibility of selective inhibition of components of the pathway to specifically affect and inhibit a virus while leaving the host cell intact.

## Data Availability

No new data were created or analyzed in this study. Data sharing is not applicable in this article.
